# A Lack of Sexual Dimorphism in Width-to-Height Ratio in White European Faces Using 2D Photographs, 3D Scans, and Anthropometry

**DOI:** 10.1371/journal.pone.0042705

**Published:** 2012-08-07

**Authors:** Robin S. S. Kramer, Alex L. Jones, Robert Ward

**Affiliations:** School of Psychology, Bangor University, Bangor, United Kingdom; University of Jyväskylä, Finland

## Abstract

Facial width-to-height ratio has received a great deal of attention in recent research. Evidence from human skulls suggests that males have a larger relative facial width than females, and that this sexual dimorphism is an honest signal of masculinity, aggression, and related traits. However, evidence that this measure is sexually dimorphic in faces, rather than skulls, is surprisingly weak. We therefore investigated facial width-to-height ratio in three White European samples using three different methods of measurement: 2D photographs, 3D scans, and anthropometry. By measuring the same individuals with multiple methods, we demonstrated high agreement across all measures. However, we found no evidence of sexual dimorphism in the face. In our third study, we also found a link between facial width-to-height ratio and body mass index for both males and females, although this relationship did not account for the lack of dimorphism in our sample. While we showed sufficient power to detect differences between male and female width-to-height ratio, our results failed to support the general hypothesis of sexual dimorphism in the face.

## Introduction

Human skulls are sexually dimorphic [1]. Men have larger bodies than women, and correspondingly larger skulls. Sexual dimorphism in size is frequently taken as evidence of male intrasexual competition, whereby larger males have a selective advantage that is passed on to their male offspring [2]. Differences in skull shape, as opposed to size, might reflect a different kind of selection pressure. An analysis of adult southern African skulls from 30 men and 30 women [1] found that men compared to women had a greater facial width-to-height ratio (WHR), that is, a wider face, which cannot be attributed to dimorphism in size. Weston et al. measured the relative width to height of the upper face, that is, the facial bones below the cranium and excluding the mandible. Width of the upper face was defined as the bizygomatic width, and height by the distance between nasion and prosthion (see also [3]). Weston et al. argued that these sex differences in skull shape might result from intersexual selection pressure, so that a region of the face has evolved which highlights the distinction between men and women. Consistent with this hypothesis, a frequent claim is that face width, as well as certain kinds of aggressive behaviours, are influenced by testosterone [4]. The implication of these views is that increased WHR may correlate with levels of other masculine characteristics, even though some of these characteristics, such as aggression, might not be in themselves sexually attractive [5]. Several recent studies have investigated this link between WHR and masculine traits in men. Men with higher WHR are more aggressive ([6], [7] cf. [8], [9]), more likely to exploit the trust of others [5], and more likely to deceive and cheat [10]. Indeed, it appears that this facial measure in male CEOs even predicts their company's financial performance [11]. In contrast, WHR in women has been found to be uncorrelated with these traits [5], [6], [10].

The possibility that WHR is a readily available signal of masculinity is intriguing. However, evidence that WHR is sexually dimorphic in faces, rather than skulls, is surprisingly weak. Previous studies have found that male WHR was significantly larger in a sample of 88 undergraduate students (37 men, 51 women) of mixed ethnicities [6]. However, in another set of studies, using two larger samples of students (192 and 123 students, ethnicities unreported), WHR differences did not reach significance [10]. Finally, in a sample of 470 Turkish people, Özener [9] found a trend for WHR to be larger in female faces. Another complication is that sexual dimorphism may be expressed differently in different populations [12]. As such, the nature of sexual dimorphism in WHR is unclear, as is the value of WHR as a signal of masculinity in faces.

One issue with the above research is the reliance on photographs. Assuming that WHR is sexually dimorphic in the face, it is still necessary that this ratio can be accurately obtained from measurements of two-dimensional images. The method of measuring WHR from photographs assumes that all specimens adopt the same posture to the camera, facing straight ahead and sitting upright. If the photographed head is tilted slightly up or down (e.g., chin raised), then measures of face height, as projected in the photo, will be reduced. If the head is turned slightly to the side (e.g., presenting more of the left side of the face than the right), then measures of face width will be less. In theory, this could have important but undesirable consequences. For example, if on average men posed for photographs with their chins slightly raised, while women faced the camera more directly, then all else being equal, men would have apparently greater WHR due to an artefactual shortening of the upper face. Similarly, if men faced the camera more directly, and women tended to turn their head slightly to the side, then all else being equal, women would appear to have reduced WHR, even if no real difference existed. Therefore, although careful work can greatly mitigate these difficulties, we believe it is important to measure WHR using measures in addition to estimates from photos.

Further, WHR might not be sexually dimorphic in the face even if it is dimorphic in skulls. The major distinction between these two sources is the presence of facial tissue. Facial soft tissue depths do not seem to significantly differ in males and females [13], [14], and as such, any individual male might well have more or less than any given female. In particular, the thickness of soft tissue that overlies the zygions can range from 1.4–21.4 mm, and appears related to general body-build [15]. This may be one factor underlying the correlation between body mass index (BMI) and WHR in both males and females [16]. But while BMI shows some sexual dimorphism, with males tending to have a larger value [17], this difference may be inconsistent through the lifespan [18], and the two sexes show significant overlap [16]. Taken together, these studies may suggest that overlaying facial tissue of fat and muscle mass onto the skull might dilute WHR dimorphism. However, if BMI is concealing true WHR differences in the face, controlling for BMI should make WHR dimorphism more apparent.

In the current research, we aimed to tackle these issues in three studies. First, we investigated WHR in a large sample of facial images of a White European population, and calculated WHR from 2D photographs. Second, we investigated WHR in another sample, this time using both 2D photographs and 3D facial scans of the same participants. The 3D scans allowed us to remove any postural effects, and collecting WHR estimates from both 2D and 3D images from the same individuals gives us a check on the consistency of measurements. Third, we collected both 2D images and facial anthropometry (measurements taken directly from the face), along with participant height and weight. Again, this allowed us to establish the agreement of measures across conditions, and in addition we examined the potential influence of BMI on sexual dimorphism.

## Materials and Methods

### Ethics Statement

All of the studies reported in the current article were approved by the Ethical Governance and Approval System at Bangor University, and all participants gave written informed consent and were treated in accordance with the ethical standards expressed in the Declaration of Helsinki.

### Study 1: 2D photographs

#### Purpose

In Study 1, we investigated WHR in 2D photographs from a large White German sample using previous methods of measurement.

#### Procedure

**Figure 1:**
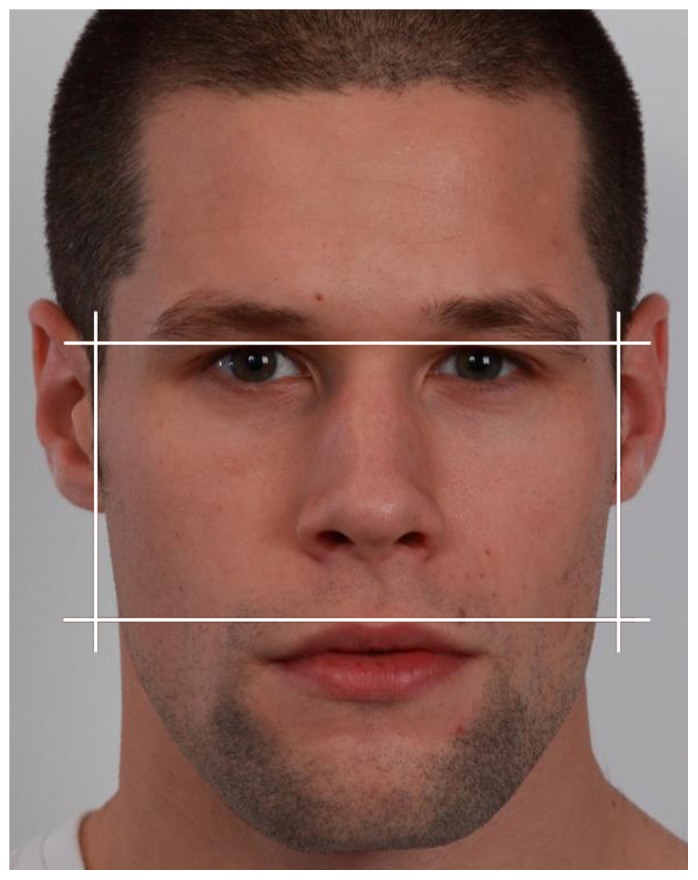
Example of relative facial width measure. An example illustrating how WHR was calculated from 2D photographs in all studies, similar to methods used by [5]–[11]. This photo is taken from Study 2. Images were rotated in order that the pupils were horizontally aligned. Facial width was measured as the horizontal distance between the left and right zygions, and height as the vertical distance between the highest point of the upper lip and the highest point of the eyelids. The WHR was calculated as width divided by height.

Four hundred and fifteen images (277 females) were downloaded from an online database (www.facity.com) that in December 2011 contained over two thousand high quality photographs of faces from cities around the world. Individuals volunteered to be photographed for the database and varied widely in attractiveness. All images were taken front-on and with neutral expressions, hair pulled back, and minimal make-up. The website is based in Germany and so photos from German cities are the most numerous. We reviewed all images from the four German cities with the largest number of photos available. Individuals were included if they had closed mouths with no visible teeth, were aged approximately 18–30 (year of birth available in the majority of cases), and were of White ethnicity as judged by the experimenters. ImageJ (NIH open-source software) was used to rotate images so that both pupils were aligned to the same transverse plane. The same software was then used to measure the width (the horizontal distance between the left and right zygion) and height (the vertical distance between the highest point of the upper lip and the highest point of the eyelids) of each image (see Figure 1). The WHR was calculated as width divided by height.

A number of studies have used a method very similar to this for measuring WHR from photographs, but it is worth being explicit about the differences between studies. [6], [7], [9]–[11] did not appear to align the pupils to the same transverse plane before measuring, and therefore defined bizygomatic width as the horizontal distance based on the frame of reference defined by the camera. If the face was slightly rotated, the width would be underestimated compared to a skull-based measure. The alignment used here has the effect of defining bizygomatic width using a face-based frame of reference. To measure height, although some previous studies have identified the “brow” as the upper landmark from facial photos [6], [7], [9]–[11], we used the top of the eyelids, similar to [5]. This has the advantage of being easily specified and agreed upon, while vertically positioned close to the nasion.

In order to determine the reliability of WHR measurement from the images, 30 randomly selected photographs were measured again by a second researcher. Agreement for WHR values across the two researchers was very high [*r*(28) = .99, *p*<.0001].

### Study 2: 2D photographs and 3D scans

#### Purpose

In Study 2, the issues of both small postural differences and potential variation in photographic methods were addressed through the use of more controlled photographic procedures, and 3D facial scanning, in order to eliminate potential postural effects.

#### Procedure

The face images used here were a subset of the database created by Jones, Kramer, and Ward [20]. Here we briefly describe the collection and production of 3D images; for full details refer to Jones et al.

Images for a group of 155 Bangor University students (89 females, aged 18–30) were selected from a larger sample on the basis of self-report as White British. Three-dimensional face measurements were captured in collaboration with Di3D (Dimensional Imaging Ltd., U.K.), using their FCS-100 system. Photographs were constrained to reflect neutral expression, eyes forward; consistent posture, lighting, and distance to the cameras; no glasses, jewellery, or make-up; and hair back. This system allows for the simultaneous capture of four images from different locations of known separation. These images are then merged using passive stereophotogrammetry software to produce high-resolution texture maps and 3D models of the participant.

The number of vertices in each facial scan was standardised by conforming each 3D model to a high resolution template containing 4735 vertices, using the Di3Dtransfer tool with a series of 48 landmarks which were manually identified on the individual 3D model and the template to increase the accuracy of the transfer. All resulting meshes had an alignment error under 0.5 mm of the original scan, meaning all vertices of the fitted meshes were within 0.5 mm of the original scan. This process ensured that each face was oriented to best fit the standardised templates.

Three-dimensional landmarks were extracted corresponding to the top of the upper eyelid fold above each eye, the top-center of the upper lip, and the lateral surface of the left and right zygions. WHR was calculated as the straight-line distance between the 3D coordinates of the zygions divided by the straight-line distance between the 3D coordinates of the eyelid (average position of the left and right) and lip landmarks.

In addition, a separate set of 2D digital photographs of each participant's face were taken by a professional photographer using a Canon EOS 5D MKII and professional-quality lighting and reflectors. Photographs were constrained as above. Landmarks were then manually identified: the left and right zygions, the highest points on the left and right eyelids, and the highest point of the upper lip. Using custom MATLAB software (The Mathworks, Natick, MA), these sets of landmarks were rotated so that both outer eye corners were aligned to the same transverse plane. The width of the faces were given by the horizontal distance between the two zygion points on the rotated image (bizygomatic width). The vertical position of the two eyelid points were averaged, and the vertical distance between this resulting value and the vertical position of the upper lip point produced the facial height. Finally, WHR was calculated as width divided by height.

### Study 3: 2D photographs and anthropometry

#### Purpose

In Study 3, we used a third technique for measuring WHR, anthropometry, where measurements are taken directly from the face. We also investigated the relationship between WHR and BMI. As noted earlier, if variation in BMI and fat tissue in the face is concealing true WHR differences, then controlling for BMI should make WHR dimorphism more apparent.

#### Procedure

From a new group of Bangor University students, 180 individuals (105 females, aged 18–29) who self-reported being White British were included in the current study. Two-dimensional digital photographs of each person's face were taken following the same procedures as in Study 2, although using a Nikon D3000. Again, landmarks were identified as before and WHR was subsequently calculated.

In addition, craniofacial measurements were obtained using Campbell 10 and 20 sliding calipers (Rosscraft, Surrey, Canada). Facial width was measured as bizygomatic width, using medium pressure during measurement that was not uncomfortable for participants. Two measures of facial height were obtained for all but three participants: nasion to the subnasale, and nasion to the highest point of the upper lip. A third measure of facial height was also obtained for 105 individuals (62 females) by asking participants to hold a popsicle stick between their teeth, and the vertical distance between this and the nasion was measured. We identified the nasion in this study, rather than the top of the eyelids, as this point falls along the midline of the face, and as such, provides a simple straight-line distance for use with calipers. We chose to explore three different landmarks for the bottom of the upper face in order to provide data comparable to both our photographic studies and Weston's [1] measures with dry skulls, given that the prosthion itself is not accessible to calipers. Finally, height and weight were measured while participants were clothed but with shoes removed.

In order to determine the reliability of these measurements, 40 randomly selected individuals were measured again by a second researcher using the same calipers. Agreement (correlations in brackets) was high for bizygomatic width (.86), nasion-subnasale (.64), nasion-upper lip (.73) (all *p*s<.0001). The nasion-stick distance was only remeasured in 21 individuals, but also demonstrated high test-retest reliability (*r*(19) = .85, *p*<.0001).

## Results

### Study 1 Results

Female WHR did not differ from male WHR (*t*(413) = 1.33, *p* = .185) (see Table 1). Technically, facial distances and ratios are not normally distributed since values cannot extend below zero. Therefore, for this and all subsequent analyses, non-parametric permutation tests were also carried out. In all cases, the results were not different from those reported here. Using the same methods as previous research, we found no sexual dimorphism in facial WHR. Although not statistically significant, women's WHR was slightly higher than men's in this sample, in line with the results of Özener [9].

**Table 1:** A summary of the three studies.

			Width-to-height ratio		
	Sex	Sample size	2D photographs	3D scans	Anthropometry*
Study 1	Male	138	1.85 (0.11)	-	-
	Female	277	1.87 (0.11)	-	-
Study 2	Male	66	2.01 (0.16)	1.83 (0.11)	-
	Female	89	2.03 (0.14)	1.87 (0.11)	-
Study 3	Male	75	2.07 (0.16)	-	1.97 (0.17)
	Female	105	2.07 (0.15)	-	2.04 (0.16)

*Note.* Mean WHRs are reported, with standard deviations in brackets.

* The WHRs included here used the nasion to the top of the upper lip as the facial height, and were therefore most similar to the other measures in terms of the physical landmarks chosen.

As discussed above, these 2D images may have contained small postural differences that created noise in the WHR measure. In turn, this noise could be sufficient to mask any sexual dimorphism. Additionally, the photographs used here were taken by multiple photographers in multiple locations, and we cannot assume there was no variation in the photographic methods. For example, subject-to-camera distance was not specified, and this factor can influence facial appearance [19]. In Studies 2 and 3, we therefore examined WHR using photographs and other measures collected in a more controlled environment.

### Study 2 Results

The two measures of WHR, from 2D photographs and 3D measurements, conducted on the same individuals but with entirely separate procedures, showed high agreement (*r*(153) = .81, *p*<.0001). The agreement between the two measures supports the validity of each.

In the 2D photographs, female WHR did not differ from male WHR (*t*(153) = 0.66, *p* = .509) (see Table 1). In the 3D measurements, female WHR was slightly larger than male WHR (*t*(153) = 2.50, *p* = .013). The opposite direction of the difference demonstrates that this facial ratio was not larger in males in our sample, in contrast with some previous research [6].

### Study 3 Results

All three craniofacial WHR measures were highly correlated with WHR from the 2D images (all *r*s>.65, all *p*s<.0001). Again, the correlation between the photographic and direct measurements supports the validity of each. However, in the 2D images, female WHR did not differ from male WHR (*t*(178) = 0.06, *p* = .950) (see Table 1). Likewise, although the three measures of WHR produced using craniofacial measurements were highly correlated with each other (all *r*s>.89, all *p*s<.0001), none showed differences in WHR. For WHR (nasion-subnasale), female WHR (*M* = 2.59, *SD* = 0.21) did not differ from male WHR (*M* = 2.54, *SD* = 0.23) (*t*(175) = 1.38, *p* = .168). For WHR (nasion-upper lip), female WHR (*M* = 2.04, *SD* = 0.16) was significantly larger than male WHR (*M* = 1.97, *SD* = 0.17) (*t*(175) = 2.84, *p* = .005). Finally, for WHR (nasion-stick), female WHR (*M* = 1.71, *SD* = 0.14) did not differ from male WHR (*M* = 1.66, *SD* = 0.13) (*t*(103) = 1.74, *p* = .086). Taken together, these results again find no support for the hypothesis that WHR is sexually dimorphic.

We next look at the influence of BMI on male and female WHR. BMI did not differ between sexes (*t*(178) = 0.50, *p* = .620), but it was correlated with WHR in both sexes. For women, BMI strongly correlated with 2D measures of WHR (*r*(103) = .43, *p*<.0001) and all three craniofacial measures of WHR (all *r*s>.29, all *p*s<.003). For men, BMI strongly correlated with 2D measures of WHR (*r*(73) = .52, *p*<.0001) and two craniofacial measures of WHR (all *r*s>.35, all *p*s<.002). Only male WHR (nasion-stick) did not significantly correlate with BMI (*r*(41) = .25, *p* = .100). These results replicate previous evidence [16] that BMI and WHR are closely related in both sexes. Changes in BMI have already been shown to have a much greater impact on facial soft tissue depths than age and sex [21], resulting in variation in facial width [15] that may overshadow any measurable differences between the sexes in WHR.

We therefore compared WHR values in samples of restricted BMI ranges. Table 2 shows the results for WHR measures taken from the photographs. We also looked at the three craniofacial measurements, but again found no case in which men had a higher WHR than women. As such, we found no support for the hypothesis that BMI variation prevents larger male skull WHR from being observable.

**Table 2:** WHR as a function of BMI category.

BMI category	Sex	Sample size	WHR	t value
All	Male	75	2.07 (0.16)	0.063
	Female	105	2.07 (0.15)	
Normal	Male	40	2.03 (0.13)	0.518
	Female	64	2.05 (0.14)	
Overweight	Male	20	2.11 (0.14)	0.698
	Female	26	2.08 (0.14)	
Normal+Overweight	Male	60	2.06 (0.13)	0.089
	Female	90	2.06 (0.14)	

*Note.* Mean WHRs from photographs are reported, with standard deviations in brackets. Underweight and obese categories were excluded due to small sample sizes.

### Power analysis of Studies 1, 2, and 3

In none of our studies did we find a trend for greater WHR in men than women, so here we consider the power of our studies to detect genuine WHR differences from our photograph stimuli. We calculate combined power for Studies 2 and 3, collected on the same student population, and calculate power separately for Study 1. In Study 1, we estimated WHR from the photographs of 138 men and 277 women (see Table 1). With an alpha of .05, we would have a power of .95 to detect an effect size of .34 (one-tailed hypothesis that men have greater WHR than women). Given the overall standard deviation in this study of .11, that effect size corresponds to a detectable difference in WHR of .04. If we combine Studies 2 and 3, we get a similar level of power: given 141 men and 194 women and an alpha of .05, we would have a power of .95 to detect a true WHR difference of .06. We can translate unitless differences in WHR into a more imageable form. For example, for an average face length of 70 mm, the face width corresponding to WHRs of 2.00 and 2.06 would be 140 and 144 mm, respectively, that is a difference of 4 mm.

The power of the studies is therefore comparable to the dimorphism in skulls reported by Weston et al. [1]. The difference in WHR based on the skull data from Weston et al. was 1.92 (WHR for men)−1.84 (WHR for women) = .08. This .08 difference in WHR can again be put in a more imageable form. For an average face height of 70 mm, the face widths corresponding to WHRs of 1.92 and 1.84 would be 134 mm and 128 mm, or a width difference of about 6 mm.

Given what looks to be sufficient experimental power, the significant correlations for different WHR measurements on the same individuals, and the consistent pattern of the studies, we have reasonable confidence that the faces of the men in our sample did not have greater WHR than those of the women.

### Comparison of direct measures: skulls, scans, and anthropometry

**Figure 2:**
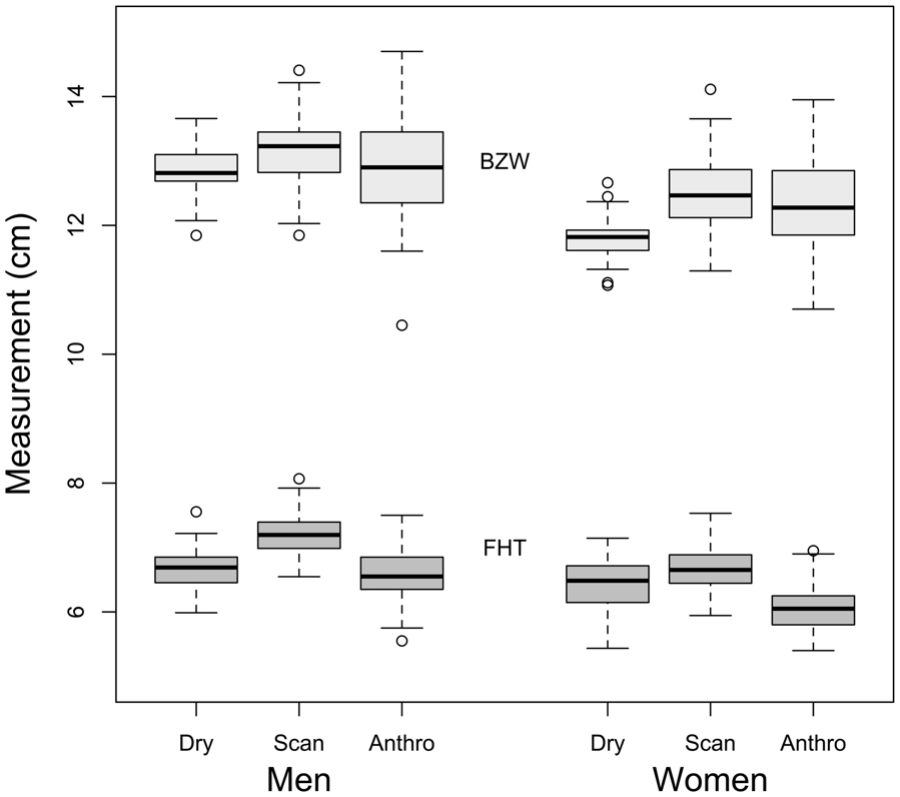
Boxplots comparing different direct measures of skulls and faces. A comparison of facial height and width measures across three samples. Dry = measurements taken from 30 male and 30 female adult southern African dry skulls, as reported in supplementary data of Weston et al. [1]; Scan = measures from the 3D scans in Study 2; Anthro = measures from the direct craniofacial measurements in Study 3. The light gray boxes indicate measures of bizygomatic width (BZW); dark grey bars are measures of face height (FHT, nasion to prosthion).

Unlike photographs, but like measurements from skulls, the 3D facial scans (Study 2) and direct craniofacial measurements (Study 3) provide absolute measures of face width and height, as well as the relative measure of WHR. Figure 2 shows the similarity in the height and width measurements of all methods. The overall similarity provides convergent validity for these different measurements, especially considering that the Weston et al. [1] sample and ours come from different racial groups and backgrounds (Black southern African and White British), and completely different methods were used to make the facial measurements in the three cases (direct from dry skull, direct from face, 3D scan).

However, to understand possible differences between WHR in faces and in skulls, it would be very valuable to have replications of WHR dimorphism in the skull in other samples, for example, using modern magnetic resonance images. The data from Weston et al. [1] are important as a measure direct from the skull, but still it is a relatively small sample, and does not show the range of variation we might expect. For example, as illustrated in Figure 2, male and female skulls showed different width, but no overall difference in height. Weston et al. argue that WHR dimorphism is driven by male and female growth trajectories that appear to diverge at puberty for facial width but not for facial height. Whether such a pattern is expected is not entirely clear. Our measures agree with sources demonstrating that both face height and width are larger in men than women, in a variety of ethnic groups [22]–[24]. In our sample, we see that men relative to women have greater facial height and width, but an equivalent WHR. More investigation into this discrepancy is clearly required.

## Discussion

In three studies, we found no evidence of sexual dimorphism in facial WHR using several methodologies and three separate samples taken from White populations in Germany and the UK. Study 1 replicated previous methods of measurement using a large sample of 2D photographs, but found no difference between men and women. Study 2 measured WHR from standard photos and also from 3D face scans, to remove any postural effects that could affect 2D measurements. Although we found good agreement between WHR computed from both sources, we did not find that men had greater WHR than women. Study 3 found no WHR differences between males and females from either standard photos or from anthropometry, although WHR values from these methods strongly correlated with each other.

We replicated previous findings of a relationship between BMI and WHR in both men and women [16]. It seems likely that BMI and other variations in the thickness of soft facial tissue will make WHR dimorphism more difficult to find in living faces than skulls, and systematic sex differences in BMI could also systematically alter WHR. However, it did not appear that BMI was concealing dimorphism in our sample. We did not see BMI dimorphism in our sample (although it is observed in other cases [17]), and perhaps more important, we did not find WHR dimorphism even when we restricted our analyses to specific BMI ranges.

Interestingly, there was a trend for females to have a larger WHR than males in some of the measures, and this even reached significance in a few cases. Özener [9] also found a nonsignificant trend for women to have larger WHR than men in a Turkish student population. While it is not clear why women may show a larger WHR, it is possible that this may reflect modern trends in BMI between the sexes. In any case, how greater WHR may relate to behaviours in women remains unknown [25].

We did not investigate the relationship between WHR and aggression, exploitation, or other masculine traits. However, previous findings demonstrating within-sex correlation between men's WHR and behaviours, such as aggressiveness, have frequently claimed that WHR is sexually dimorphic [6], [7], [10], [11]. A possible implication – one that is not drawn by the authors above, but which might be easy to slip into – is that factors explaining dimorphism in morphology and behaviour (e.g., sex-typical hormones) may also be responsible for any within-sex correlations between men's WHR and behaviour. However, our results and others [9] suggest that such a conclusion would not be based on safe assumptions. It may be more fruitful for researchers investigating the correlations between facial appearance and behaviour to focus on other characteristics, such as the jaw and brow, which are clearly dimorphic [26].

Our findings speak more directly to the hypothesis of Weston et al. [1], that dimorphism in facial width is the result of sexual selection, with women favouring men with wide faces. There is no reason to assume that women's mate choice would be biased to create a dimorphism that does not exist. We favour the view of Stirrat and Perrett [5], who found that relatively wide men's faces were rated as unattractive (as would be the correlated untrustworthiness), and on that basis suggest that women's mate choice would, if anything, select against wide faces. Variation in men's facial width might then be maintained by the conflicting pressures of sexual selection and intrasexual competitive displays.

Finally, it seems perfectly possible that skull differences on the order of several millimetres could still carry important signal content. But ultimately, a visual signal need not be on the skull but must be on the face. If we assume that WHR in the skull did confer an advantage for sexual selection, then it would not be surprising to find soft tissue deposits used to mimic the signal, and exaggerate the fitness display. Soft tissue could therefore mask or exaggerate potential WHR signals from the skull, leading to a breakdown of the signal system [27].
